# Common genetic polymorphisms define one-carbon metabolite responses to different forms of choline in healthy adult males

**DOI:** 10.3389/fnut.2025.1620538

**Published:** 2025-12-03

**Authors:** Nisa Butt, Jianzhang Dong, Gia V. Shelp, Elizabeth M. Poole, Justine R. Keathley, Marica Bakovic, Clara E. Cho

**Affiliations:** 1 Department of Human Health Sciences, University of Guelph, Guelph, ON, Canada; 2 Department of Family Relations and Applied Nutrition, University of Guelph, Guelph, ON, Canada

**Keywords:** choline, phosphatidylcholine, single nucleotide polymorphisms, one-carbon metabolism, multivariate analysis

## Abstract

**Background:**

Responsiveness to nutrients can be determined by many types of variations, such as single-nucleotide polymorphisms (SNPs). Choline is an essential nutrient critical for proper organ function and exists in different forms, such as free choline or as derivatives, including phosphatidylcholine (PC). Although genetic variations in genes encoding enzymes that influence choline metabolism have been identified, little is known regarding individual responses to free choline and PC in relation to SNPs. Here, we determined the effect of different forms of choline, genotype, and their interaction on one-carbon metabolite concentrations in urine, which has utility in capturing the overall change in choline metabolism.

**Methods:**

A randomized, double-blinded, crossover study was conducted in healthy adult males (*n* = 37) who were provided with a standardized meal containing 600 mg choline, either as choline bitartrate (CB) or PC, or no choline (NC). Urine was collected at study baseline and pooled throughout the 6-h study duration. *Choline dehydrogenase* (*CHDH*) rs12676, *betaine-homocysteine S-methyltransferase* (*BHMT*) rs3733890, *choline kinase alpha* (*CHKA*) rs10791957, and *phosphatidylethanolamine N-methyltransferase* (*PEMT*) rs4646343 were genotyped.

**Results:**

There was a main treatment effect for urinary choline change from baseline, reflective of differences in absorption by free choline versus PC (*p* < 0.01). A reduction in responsiveness to CB was found with genetic variation in *CHDH* rs12676, manifested as lower choline oxidation (*p* < 0.05), and downstream pathways in the methionine cycle (*p* < 0.01), whereas a reduction in responsiveness to PC occurred with genetic variation in *BHMT* rs3733890 (*p* < 0.05). Genetic variations in *CHKA* rs10791957 and *PEMT* rs4646343 reflected differences in the partitioning of choline in response to CB and PC (*p* < 0.01). Multivariate analysis showed that groups with an accumulated number of effect alleles across all SNPs have contrasting responses to CB and PC that deviate from the patterns derived from treatment effect alone (*p* < 0.05).

**Conclusion:**

Unique metabolite signatures in one-carbon metabolism arise in response to supplemental intake of different forms of choline, driven by genetic variations that regulate choline homeostasis. Our findings highlight the importance of nutrient–gene interactions in deciphering the complexity of individual metabolic responses, supporting the emerging concept of precision nutrition.

## 1 Introduction

Current dietary recommendations are predominantly centered on a “one-size-fits-all” approach, but they ignore the fundamental principle that responses to nutrients and their requirements are highly individualistic (1). Precision nutrition, often grouped with personalized nutrition, is an emerging field that considers the sources of metabolic variations to help target specific treatments for specific groups of individuals for optimal health (2). There are many inputs that contribute to metabolic differences at the individual level, such as genetic, epigenetic, gut microbiome, and lifestyle factors (3). Among these, genetic variations have been studied extensively, particularly single-nucleotide polymorphisms (SNPs) (2, 4), which are single base-pair substitutions in genes that can alter enzyme function and influence nutrient metabolism.

**Figure 1:**
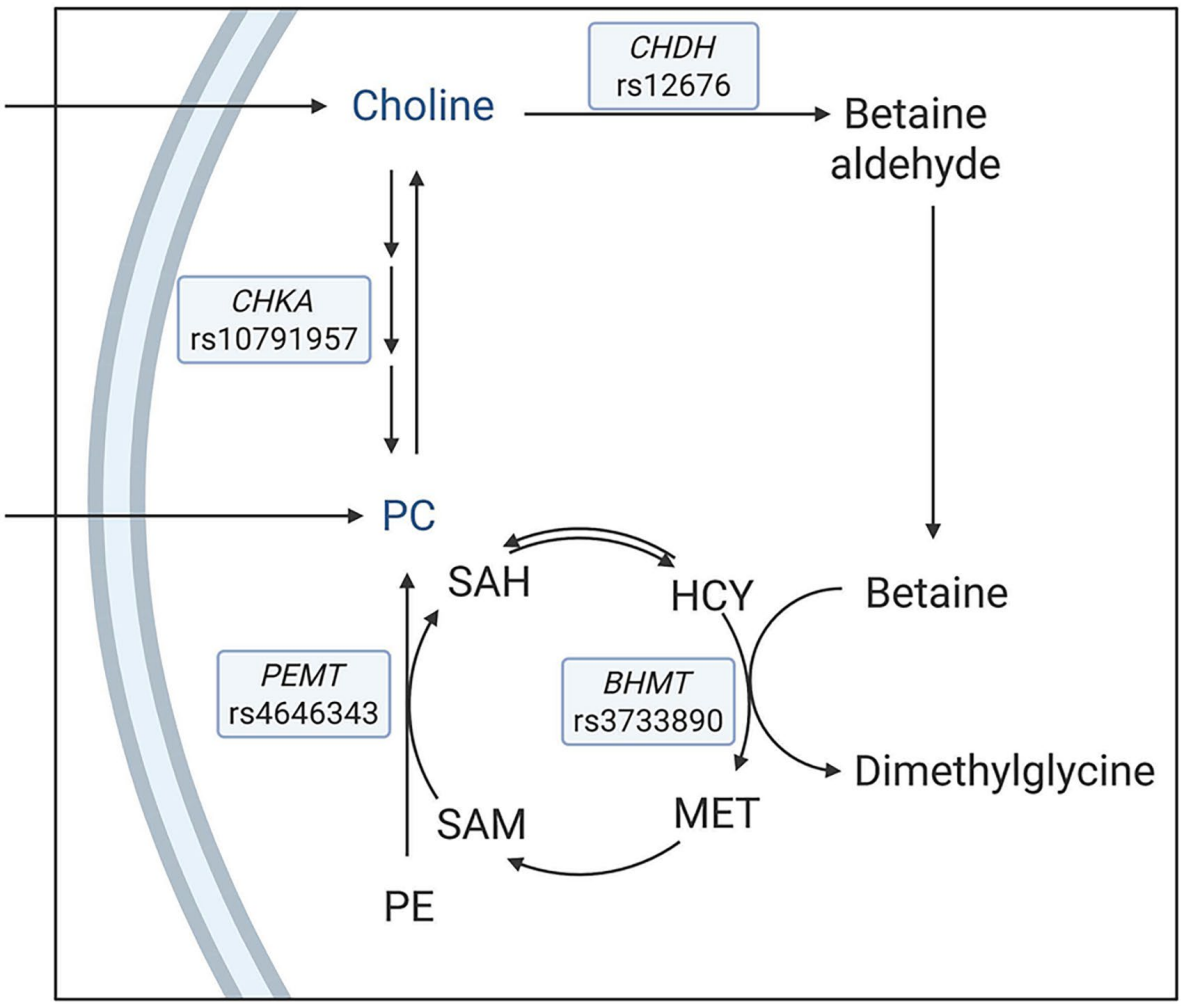
A simplified schematic of metabolic pathways with a focus on enzymes that influence choline metabolism. The single nucleotide polymorphisms under investigation were *choline dehydrogenase* (*CHDH*) rs12676, *betaine:homocysteine methyltransferase* (*BHMT*) rs3733890, *choline kinase A* (*CHKA*) rs10791957, and *phosphatidylethanolamine N-methyltransferase* (*PEMT*) rs4646343. HCY, homocysteine; MET: methionine; SAM: *S*-adenosylmethionine; SAH: *S*-adenosylhomocysteine; PE: phosphatidylethanolamine; PC: phosphatidylcholine.

SNPs can modulate metabolic pathways through gene–nutrient interactions, offering valuable insights into individualized dietary requirements and the biological diversity of nutrient responses (5). Choline serves as a key example in which metabolic pathways are under the regulation of SNPs. As a precursor for several biomolecules, choline exerts a wide range of biological effects, such as cholinergic neurotransmission, cell membrane composition and signaling, lipid transport, and the provision of methyl groups within the interconnected biochemical network of pathways called one-carbon metabolism (6–8). Various functional SNPs in genes encoding enzymes involved in choline metabolism have been identified, indicating their impact on choline homeostasis (9, 10) (Figure 1).

In one route, choline is irreversibly oxidized to betaine via choline dehydrogenase (CHDH) and betaine aldehyde dehydrogenase. Betaine then donates a methyl group to homocysteine to form methionine via betaine:homocysteine methyltransferase (BHMT), and, in turn, is converted to dimethylglycine. Methionine is used for the biosynthesis of *S*-adenosylmethionine, a universal methyl donor for various acceptor molecules, including DNA, phospholipids, and proteins, and is subsequently converted to *S*-adenosylhomocysteine (11). *CHDH* rs12676 (A → C) is suggested to reduce the activity of CHDH, potentially limiting betaine production and impairing methyl group availability (12). *BHMT* rs3733890 (G → A) affects the methyl transfer from betaine to homocysteine, with the A allele associated with reduced methylation capacity (13).

In a separate pathway, choline is phosphorylated via the cytidine diphosphate (CDP)-choline pathway to synthesize phosphatidylcholine (PC), with choline kinase A (CHKA) as the first enzyme in the pathway (14). Additionally, the *de novo* pathway via phosphatidylethanolamine *N*-methyltransferase (PEMT) catalyzes the sequential methylation of phosphatidylethanolamine using *S*-adenosylmethionine-derived methyl groups to synthesize PC, which can be converted to choline (15, 16). *CHKA* rs10791957 (C → A) contributes to the modulation of PC homeostasis, in which the A allele has been shown to direct more dietary choline to the CDP–choline pathway relative to the PEMT pathway (10). *PEMT* rs4646343 (G → T) influences the activity of PEMT, with the T allele associated with lower endogenous PC production (17).

Although the endogenous production of choline is possible in humans, this amount is insufficient to meet physiological demands and therefore must be obtained through diet (18). The Institute of Medicine (now the National Academy of Medicine) in the United States established the adequate intake (AI) for choline at 550 mg/day for men and 425 mg/day for women (19), whereas the European Food Safety Authority set the AI at 400 mg/day for adults (20). However, choline intake has been reported to be insufficient for various population groups globally, with the average choline intake among adults estimated to be 293 mg/day (non-European countries) and 310 mg/day (European countries) (21). In light of this gap, recent discussions have emphasized the potential need for additional intake of choline through diet or supplementation to achieve the AI recommendations (22, 23).

Dietary choline exists in multiple forms, including free choline and PC, each with distinct absorption and metabolic profiles (24). Free choline is rapidly absorbed in the small intestine by enterocytes via a carrier-mediated transport process (25). In contrast, PC requires a series of hydrolysis in the intestinal lumen by pancreatic phospholipase A2 to produce lysophosphatidylcholine, which can be taken up by enterocytes, resynthesized into PC, and incorporated into chylomicrons for transport through the lymphatic system or further be hydrolyzed to glycerophosphocholine, and ultimately to glycerophosphate and free choline (26). Thus, these differences suggest that free choline is more rapidly absorbed than PC and may have distinctive effects on subproducts of choline, including those in one-carbon metabolism. Building upon this, our recent focus was on the metabolic differences between choline forms (27), with an initial series of studies investigating the impact of choline intake on the production of gut microbiota-associated metabolites (28, 30). However, the role of genetic variation in modulating individual responses to free choline versus PC, particularly in the context of one-carbon metabolism, remains poorly understood. Further, gene–nutrient interactions are often examined in isolation, limiting their capacity to reflect the full complexity of these relationships. To fully incorporate the interplay among multiple SNPs, advanced multivariate modeling, such as principal component analysis and permutation testing, can help refine subgroup classifications and inform precision nutrition strategies.

**Figure 2:**
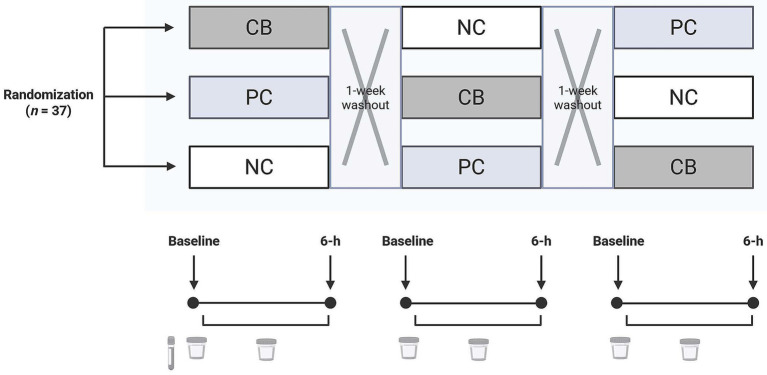
A diagram of the study design in which participants (*n* = 37) were randomized and double-blinded to receive a standardized meal containing 600 mg choline either as choline bitartrate (CB) or phosphatidylcholine (PC), or no choline (NC) control, with a 1-week washout break in between. For each study session, participants arrived after a 10-h overnight fast. Fasting blood at the first study baseline was used to isolate the buffy coat for genotyping. Urine was collected at study baseline and throughout the 6-h study period for quantification of one-carbon metabolites.

Thus, this current study represents the first to determine genotype-specific effects of choline form on one-carbon metabolism using both individual SNP and multivariate analysis. Through this framework, we were able to assess not only the isolated impact of each SNP but also its combined influence on metabolic outcomes, offering an integrative view of gene–nutrient interactions. We targeted four functional SNPs (*CHDH* rs12676, *BHMT* rs3733890, *CHKA* rs10791957, and *PEMT* rs4646343), each representing a regulatory point in choline oxidation, methylation (via betaine), phosphorylation, and synthesis of the choline moiety (10). Using a randomized double-blinded crossover study design that provided free choline versus PC, compared to a no choline (NC) control, we measured changes in metabolite concentrations pooled over a 6-h study period. This approach has the utility in capturing the average rather than different parts of kinetics and serves as a sensitive marker of response variables in one-carbon metabolism. We hypothesized that responsiveness to free choline versus PC is diminished by SNPs in genes encoding key enzymes in one-carbon metabolism, with genotype-dependent differences that classify distinct metabolic subgroups. The objective of this research was to determine the effect of choline form, genotype, and their interaction on metabolite signatures of one-carbon metabolism.

## 2 Materials and methods

### 2.1 Study design and participants

This study was a *post hoc* analysis from a parent trial (27) with a revised sample size calculation of *n* = 37, providing 80% power to detect a 10% difference in choline-derived metabolite concentrations across three groups using a repeated measures design accounting for a 10% anticipated dropout rate. However, because the parent trial was not originally designed to assess genotype effects, our analyses contained, as expected, unequal genotype distributions and necessitated pooled comparisons, specifically grouping individuals with none or one copy of the variant allele versus individuals with two copies of the variant allele. We estimated that *n* = 10 per genotype group would provide 80% power to detect at least a 12% difference in choline concentration on the basis of a within-group variance of 1.1 and *α* = 0.05. Healthy adult males (*n* = 37) consumed 600 mg choline either as choline bitartrate (CB; Balchem, Montvale, NJ, USA) or as PC (Alcolec 40P; American Lecithin Company, Oxford, CT, USA), or NC in controls in a randomized, crossover study (27) (Figure 2). Each meal was administered within a single day with a 1-week washout break. Participants were free-living, without any chronic diseases, and had not taken probiotics, prebiotics, or antibiotics for 2 months leading up to the start of the study.

For each study session, participants arrived after a 10-h overnight fast, and a baseline blood sample was drawn by a phlebotomist using venipuncture. Participants were asked to collect their baseline urine in a wide-mouth specimen container (Thermo Fisher Scientific, Wilmington, DE, USA). Then a study meal was provided, either a choline supplement powder or no powder, which was mixed into one cup (237 mL) of tomato soup and served with a bagel with margarine–butter spread and one cup of water. Participants consumed the study meal within a 15-min duration. Following the meal consumption, participants were asked to collect their urine in a wide-mouth polyethylene bottle (Nalgene) throughout the 6-h study period. At 4.5 h, a fixed snack (apple sauce) and water were provided. During each study session, participants refrained from consuming food or beverages outside those provided by study personnel.

Participants and study personnel, including those who analyzed the samples, were blinded from the order of the meal. Participants were recruited from Logan, UT, USA, and the surrounding areas. Written informed consent was obtained from each participant in the study. The experimental procedures were approved by the Institutional Review Board at Utah State University, and the study was registered with clinicaltrials.gov (NCT04255368). Sample analyses were conducted at the University of Guelph.

### 2.2 Study design justification

The chosen dose of 600 mg/day is within the physiologically relevant range and well below the Tolerable Upper Intake Level of 3,500 mg/day (19). A single meal ingestion employed in this study is advantageous in capturing the acute metabolic response, coupled with a crossover design that serves to minimize unaccounted differences in personal characteristics in a parallel arm design. The larger study was intended to include adult males and females as well as two distinctive categories of BMI to examine the effect of sex and BMI on metabolite responses, but there was an insufficient number of females and a higher BMI category, leading to a *post hoc* analysis that included only males and pooled samples of all BMI categories.

### 2.3 Sample collection

Baseline venous blood samples were collected in EDTA tubes, placed on ice and then centrifuged at 2,000×*g* at 4 °C for 10 min. The buffy coat layer was isolated and transferred into cryogenic tubes containing DMSO and then mixed by inversion. Baseline and 6-h study urine in collection containers were kept on ice, gently mixed, and then transferred into cryogenic tubes. All aliquoted samples were immediately stored at −80 °C until further use.

### 2.4 Urinary choline, betaine, dimethylglycine, and methionine concentrations

Liquid chromatography tandem mass spectrometry (LC–MS/MS) was used to quantify urinary concentrations of free choline, betaine, dimethylglycine, and methionine, as previously described (29) with modifications (30, 31). Choline was measured separately using a Surveyor HPLC system (Thermo Fisher Scientific) interfaced with a TSQ Quantum Ultra Mass Spectrometer (Thermo Fisher Scientific). Betaine, dimethylglycine, and methionine were measured using a UHPLC Thermo Ultimate 3000 (Thermo Fisher Scientific) coupled to an EVOQ Qube Triple Quadrupole Mass Spectrometer (Bruker Daltonics, Bremen, Germany). The mass spectrometer was operated using electrospray ionization in positive ion mode.

Sample preparation was performed by mixing 50 μL of urine with 100 μL of acetonitrile, 0.1% (v/v) formic acid, and isotope-labeled internal standards, including d13-choline (D-5185; CDN Isotopes, Pointe-Claire, QC, Canada) or with d3-betaine (D-6303; CDN Isotopes), d3-dimethylglycine (D-7024; CDN Isotopes), and 13C-methionine (A39248; Thermo Fisher Scientific). The mixture was vortexed and centrifuged at maximum speed for 5 min. The resulting supernatant was transferred to glass vials with disposable inserts and then diluted 1:1 with a solution of water:acetonitrile:formic acid in a ratio of 1:1:0.0005.

A 10 μL aliquot of the supernatant was injected onto a Prevail Silica analytic column (150 × 2.1 mm, 5 μm particle size; Grace, Columbia, MD, USA) with a matching guard column. The mobile phase consisted of 81% acetonitrile and 19% 15 mmol/L ammonium formate with 0.1% (v/v) formic acid. The flow rate was maintained at 500 μL/min, with the column and autosampler temperatures set at 25 °C and 5 °C, respectively. Calibration curves were constructed by diluting known concentrations of choline, betaine, dimethylglycine, and methionine in water.

The parent ion/daughter ion fragments were monitored in multiple-reaction monitoring mode to detect the following transitions: *m/z* 104.2 to 60.2 for choline, *m/z* 117.2 to 69.2 for d13-choline; *m/z* 118.2 to 58.4 for betaine, *m/z* 121.2 to 61.4 for d3-betaine; *m/z* 104.2 to 58.4 for dimethylglycine, *m/z* 107.2 to 61.4 for d3-dimethylglycine; and *m/z* 150.1 to 104.1 for methionine, *m/z* 151.1 to 105.1 for 13C-methionine. The intra- and inter-assay percent coefficients of variation were 4% and 6% for choline, 3% and 4% for betaine, 5% and 6% for dimethylglycine, and 3% and 6% for methionine, respectively. The data were acquired using XCalibur software (Thermo Fisher Scientific) for choline measurements, and Compass HyStar (Bruker Daltonics) for betaine, dimethylglycine, and methionine.

### 2.5 Urinary creatinine determination

Creatinine concentrations in urine were measured using a Creatinine (urinary) Colorimetric Assay Kit (#500701; Cayman Chemical; Ann Arbor, MI, USA) based on the Jaffe reaction that involves alkaline picrate treatment. The corrected absorbance was derived from the initial and final absorbance before and after acidification at 500 nm using an Azure Ao Microplate Reader AC3000 (Azure Biosystems, Dublin, CA, USA). Creatinine concentrations were calculated using the linear regression of the standard curve by substituting corrected absorbance and were used to adjust urinary one-carbon metabolite concentrations.

### 2.6 End-point genotyping

Genomic DNA was isolated from the buffy coat of blood samples at study baseline using the DNeasy Tissue kit (Qiagen, Hilden, Germany) according to the manufacturer’s protocol. The quantity and quality of genomic DNA samples were measured using an Epoch Spectrophotometer (Agilent BioTek, Santa Clara, CA, USA). PCR reactions composed of 20 ng DNA, and TaqMan SNP Genotyping Assay Mix (Thermo Fisher Scientific) and TaqMan Genotyping Master Mix (Thermo Fisher Scientific) were performed using Endpoint Genotyping on an Applied Biosystems QuantStudio 7 System (Thermo Fisher Scientific) in the Advanced Analysis Centre Genomics Core at the University of Guelph. The chosen markers *CHDH* rs12676, *BHMT* rs3733890, *CHKA* rs10791957, and *PEMT* rs4646343 represent functional variants that are known to influence choline homeostasis and are listed in Supplementary Table 1. All samples were run in duplicate reactions with in-run standards and negative water controls with the following conditions: 95 °C for 10 min, followed by 40 cycles of 95 °C for 15 s, and 60 °C for 1 min. Pre- and post-PCR reads were analyzed using Design & Analysis Software 2 version 2.7.0 (Thermo Fisher Scientific).

### 2.7 Statistical analysis

SAS Version 9.4 (SAS Institute Inc., Cary, NC, USA) and MetaboAnalyst 6.0 (32) were used to analyze the data. A *Χ*^2^ test was used to assess whether genotype frequencies were in Hardy–Weinberg equilibrium (33). Normality and homogeneity of the data were examined using the Shapiro–Wilk test and Levene’s test, respectively, and Q-Q plots were used for visual inspection. Each metabolite was expressed as a change from baseline, where the concentration values of pooled urine throughout the 6-h study period were subtracted from the study baseline at 0 min. A mixed model was employed using PROC MIXED with treatment, genotype, and their interaction terms, followed by *post hoc* pairwise comparisons adjusted using the Tukey–Kramer method. The subject was treated as a random effect.

Multiple types of variation are expected to arise with each experimental factor (34), as this was a multifactor study. Thus, a multivariate analysis approach is considered valuable when it reduces complex patterns into components, from which the proportion of the total variation explained by each experimental factor can be identified (35). To investigate the combined effects of SNPs as their overall contribution, SNPs were coded as 0, 1, or 2, representing the number of variant alleles (0 having no copy of the SNP; 1 having one copy of the SNP; and 2 having two copies of the SNP). Given the exploratory nature of our study and the potential for multiple SNPs to exert graded effects on choline metabolism, we prioritized the additive genotype model as a biologically plausible question. While dominant and recessive models can offer increased power under specific assumptions in genetics, they may impose constraints that obscure intermediate effects derived from heterozygosity. Thus, we coded genotype information as 0-1-2 based on its ability to preserve allelic dosage information, which aligns with our mechanistic interest in gene–nutrient interactions. Thus, a polygenic score was derived by summing the number of copies of the SNP for all four genotypes of interest. The relative frequency of individuals in different bins of polygenic score was expressed as a histogram.

The metabolite data were log-transformed and range-scaled (mean-centered and divided by the range of each variable) for multivariate analysis. Principal component analysis (PCA) was conducted, in which data matrices were visualized in two-dimensional scatter plots of samples projected onto pairs of principal components. Permutational multivariate analysis of variance (PERMANOVA; 999 permutations) was applied to the sample scores of each principal component. We utilized ANOVA simultaneous component analysis (ASCA), a multivariate extension of univariate ANOVA (36), using the two-factor design module within MetaboAnalyst. Our decision to use ASCA was guided by the specific analytical priorities of our study. Generalized linear mixed models (GLMMs) as well as repeated measures ASCA+ are suitable for formal inference of longitudinal designs, but may be prone to overparameterization or lack of convergence in high-dimensional settings with modest sample sizes. We are aware that GLMMs are generally more appropriate than ASCA-based methods, as GLMMs offer flexibility in handling imbalances in the dataset, missing data, and within-subject correlations. Our dataset was complete with no missing values, and polygenic scores were treated as categorical predictors with approximately symmetric distribution. We acknowledge that genotype group sizes at the SNP level reflected allele frequencies, but this was not due to sampling artifacts. Subject-level information was incorporated as a within-subject factor, all of which represented a pragmatic approach for complex modeling. We note that ASCA is particularly well-suited for decomposing multivariate data into effect-specific matrices and applies PCA to each, thereby revealing covariance patterns among variables within each factor. In addition, reliable estimates of factor-level effects are achieved through ASCA with intuitive visualizations that support biological insight. This facilitates interpretation of coordinated biochemical responses, rather than isolated univariate effects or outcome prediction. Overall, ASCA provided the optimal balance of interpretability and strength in multivariate decomposition, aligning with our primary objective of identifying drivers of metabolic variations among groups of individuals. The total variation was partitioned into main treatment and genotype effects and their interaction. Principal component was applied to each effect matrix, and a scree plot was used to evaluate the contribution of each component to total variance. The component explaining the highest proportion of variance was extracted (in our case, component 1) to identify dominant patterns. To validate model significance, permutation tests (100 permutations) were employed within ASCA using Manly’s approach (37), which recalculates the total sum of squares for each factor and their interactions. An effect was considered significant if the observed sum of squares exceeded the 95th percentile of the permuted distribution. The Benjamini–Hochberg false discovery rate adjustment was considered to correct PERMANOVA *p*-values. Statistical significance was declared at *p* ≤ 0.05. All data are expressed as mean ± SEM unless stated otherwise.

## 3 Results

### 3.1 Participant characteristics and genotype distribution

Participants (*n* = 37) in the study had an average age of 26.3 ± 0.8 years and a BMI of 26.4 ± 0.8 kg/m^2^. The number of participants in each genotype is shown in Table 1 and conformed to Hardy-Weinberg equilibrium. The allele frequency distribution was 0.770 for the C allele of *CHDH* rs12676; 0.608 for the A allele of *BHMT* rs3733890; 0.581 for the A allele of *CHKA* rs10791957; and 0.419 for the T allele of *PEMT* rs4646343 (Table 1). Our interest was to compare individuals who are carriers of two copies of the variant allele compared to all other groups, thus, individuals with the non-variant and one variant copy carriers were pooled together.

**Table 1:** Genotypic distribution of participants across single nucleotide polymorphisms (SNPs) in genes encoding key enzymes in one-carbon metabolism: *choline dehydrogenase* (*CHDH*) rs12676, *betaine:homocysteine methyltransferase* (*BHMT*) rs3733890, *choline kinase A* (*CHKA*) rs10791957, and *phosphatidylethanolamine N-methyltransferase* (*PEMT*) rs4646343.

Gene and SNP	Genotype distribution			Allele frequency	*p*-value	Study comparisons	
						Individuals with none or one copy of the variant allele	Individuals with two copies of the variant allele
*CHDH* rs12676	AA	AC	CC	C allele frequency	NS	AA + AC	CC
	4	9	24	0.770		13	24
*BHMT* rs3733890	GG	GA	AA	A allele frequency	NS	GG + GA	AA
	8	13	16	0.608		21	16
*CHKA* rs10791957	CC	CA	AA	A allele frequency	NS	CC + CA	AA
	6	19	12	0.581		25	12
*PEMT* rs4646343	GG	GT	TT	T allele frequency	NS	GG + GT	TT
	15	13	9	0.419		28	9

Participant counts are reported for each genotype. All distributions conformed to Hardy–Weinberg equilibrium, as assessed by a *Χ*^2^ test. To maximize differences imposed by metabolic inefficiencies, individuals carrying two copies of the variant allele were compared against those with none or one copy of the variant allele. NS denotes not significant.

### 3.2 One-carbon metabolite response

**Figure 3:**
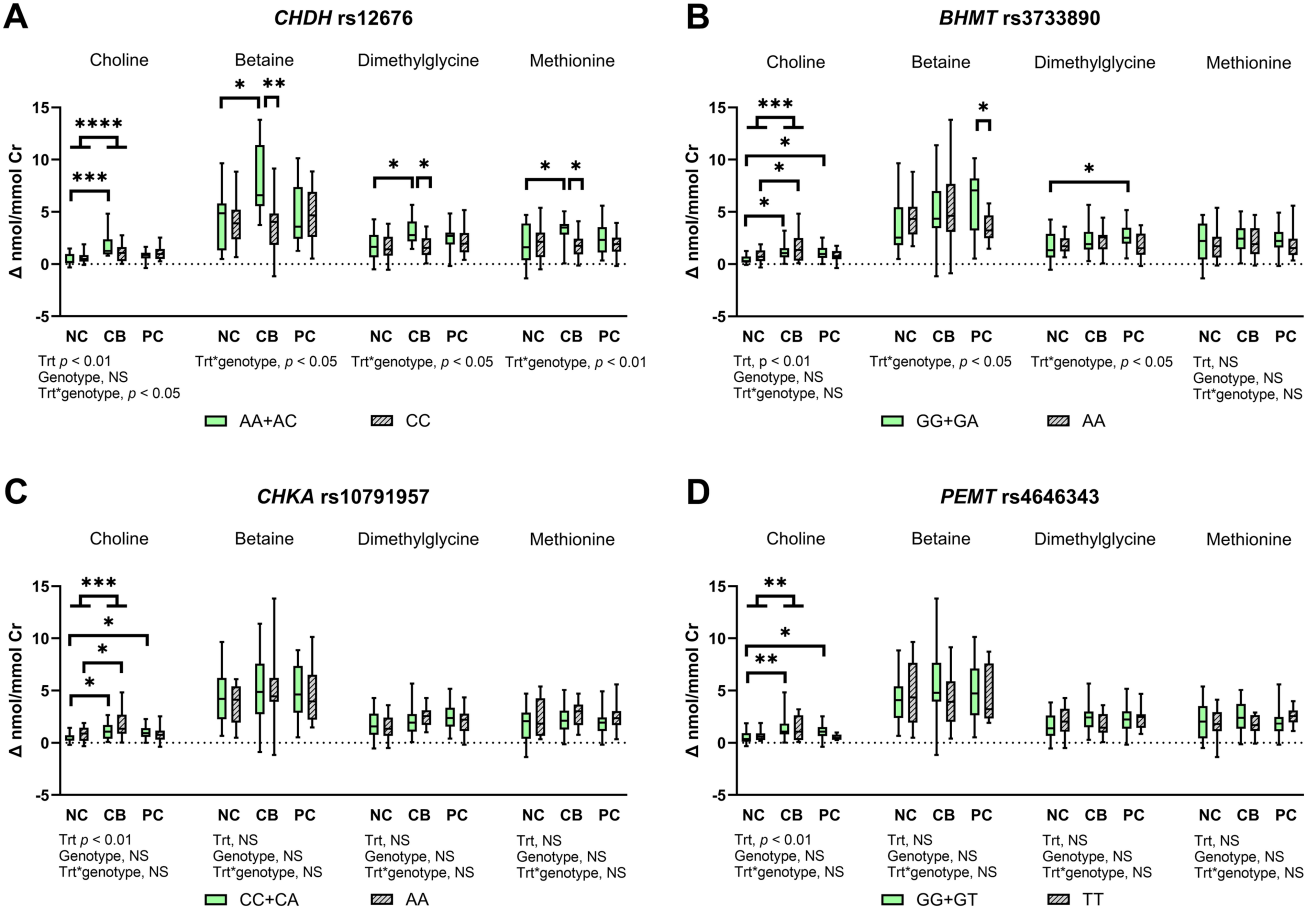
The effect of choline treatment and genetic variations on urinary concentration change from baseline for choline, betaine, dimethylglycine, and methionine after choline bitartrate (CB), phosphatidylcholine (PC), or no choline (NC) consumption in a randomized, double-blinded crossover study, shown as box and whisker plots for **(A)** *choline dehydrogenase* (*CHDH*) rs12676, **(B)** *betaine:homocysteine methyltransferase* (*BHMT*) rs3733890, **(C)** *choline kinase A* (*CHKA*) rs10791957, and **(D)** *phosphatidylethanolamine N-methyltransferase* (*PEMT*) rs4646343. Urinary metabolite concentrations were creatinine (Cr)-adjusted. The treatment (trt), genotype, and interaction effects were determined using a mixed model followed by the Tukey–Kramer *post hoc* comparisons as denoted by *p < 0.05; **p < 0.01; ***p < 0.001; ****p < 0.0001. CB yielded a higher urinary choline concentration change from baseline compared to NC, without differences in PC compared to either NC or CB. Genetic variation in *CHDH* rs12676 yielded less response to CB, whereas genetic variation in *BHMT* rs3733890 showed lower utilization of dimethylglycine in response to PC. Genetic variations in *CHKA* rs10791957 and *PEMT* rs4646343 reflected disturbances in the partitioning of choline for choline recycling and synthesis. NS denotes not significant. Box-and-whisker plots display the minimum, median and maximum values for each group, *n* = 37.

A treatment effect was present, where CB resulted in higher urinary concentration change from baseline compared to NC, whereas no difference was found between PC and NC, as well as between PC and CB (*p* < 0.01; Figures 3A–D). There was no genotype effect alone for all SNPs examined (*CHDH* rs12676, *BHMT* rs3733890, *CHKA* rs10791957, and *PEMT* rs4646343) (Figures 3A–D). Supplementary Tables 2–5 show descriptive statistics groupwise.

There was a significant treatment and *CHDH* rs12676 genotype interaction for urinary change from baseline for choline, betaine, dimethylglycine, and methionine (*p* < 0.05 for choline, betaine, and dimethylglycine, *p* < 0.01 for methionine; Figure 3A). CB induced higher urinary change from baseline for choline, betaine, dimethylglycine, and methionine compared to NC in the AA + AC genotype group only, whereas these effects did not occur in the CC genotype group. In addition, lower urinary change from baseline for betaine, dimethylglycine, and methionine was found in the CC genotype group compared to the AA + AC genotype group. No other effects, including those from PC treatment, were found.

For *BHMT* rs3733890, the GG + GA genotype group, as well as the AA genotype group, had higher choline change from baseline after CB compared to NC (*p* < 0.01; Figure 3B). The GG + GA genotype group also had higher urinary choline change from baseline after PC compared to NC, without differences between PC and NC in the AA genotype group. Treatment and genotype interacted to influence urinary betaine change from baseline (*p* < 0.05; Figure 3B), which was lower in the AA genotype compared to the GG + GA genotype group upon PC supplementation. An interaction between SNP and treatment was also detected for urinary dimethylglycine change from baseline (*p* < 0.05; Figure 3B), which was higher after PC compared to NC in the GG + GA genotype group, but absent in the AA genotype group. Urinary methionine change from baseline did not differ across groups.

For *CHKA* rs10791957, the CC + CA genotype, as well as the AA genotype groups, showed higher urinary choline change from baseline after CB compared to NC. Higher choline change from baseline after PC was observed compared to NC control in the CC + CA genotype group, but these effects were absent in the AA genotype group. No other differences occurred for urinary change from baseline for downstream products of one-carbon metabolism, including betaine, dimethylglycine, and methionine.

For *PEMT* rs4646343, the GG + GT genotype group had higher urinary choline change from baseline with CB or PC supplementation compared to the NC control. In contrast, these effects were not observed in the TT genotype group. As with the *CHKA* genotype, urinary change from baseline for betaine, dimethylglycine, and methionine did not show differences among groups.

### 3.3 Multivariate analysis

**Figure 4:**
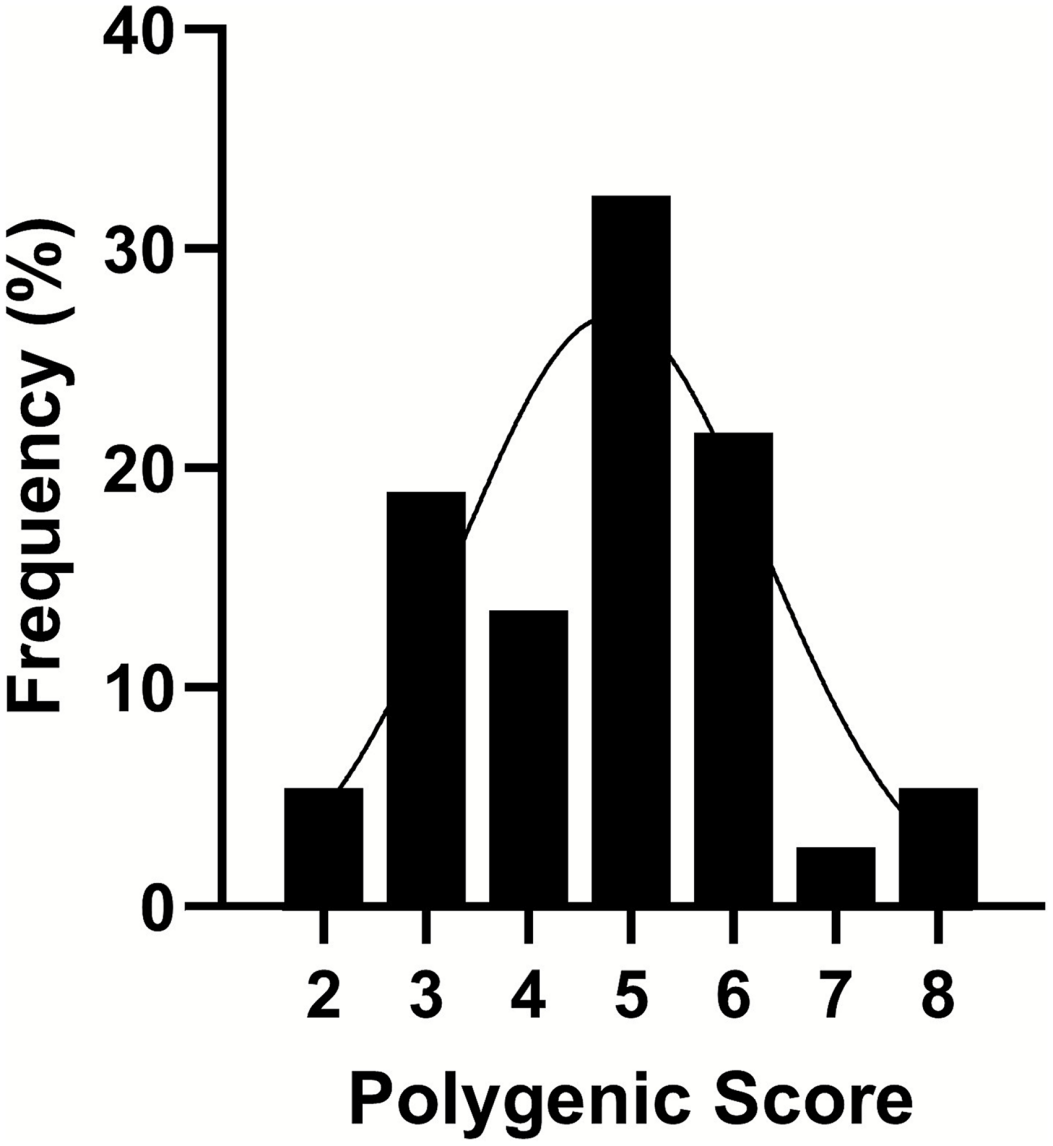
A frequency distribution of polygenic scores binned from 2 to 8 as a combined effect of the presence of the variant allele in the genotypes of *choline dehydrogenase* (*CHDH*) rs12676, *betaine:homocysteine methyltransferase* (*BHMT*) rs3733890, *choline kinase A* (*CHKA*) rs10791957, and *phosphatidylethanolamine N-methyltransferase* (*PEMT*) rs4646343. Single-nucleotide polymorphisms (SNPs) were coded as 0, 1, or 2, representing the number of variant alleles (0 having no copy of the SNP; 1 having one copy of the SNP; and 2 having two copies of the SNP). Then the polygenic score was derived from summing the number of copies of the SNP. A line shows a fitted Gaussian distribution, *n* = 37.

Our approach moved beyond the singular analysis of select SNPs and utilized a multivariate technique with dimensional reductions. We assessed the cumulative impact of SNPs using polygenic scoring that was determined by summing the number of risk alleles of each SNP across *CHDH* rs12676, *BHMT* rs3733890, *CHKA* rs10791957, and *PEMT* rs4646343. The polygenic scores were categorized as 2–8, and the relative frequency, as the proportion of individuals by different bins of scores, followed a normal distribution (Figure 4).

**Figure 5:**
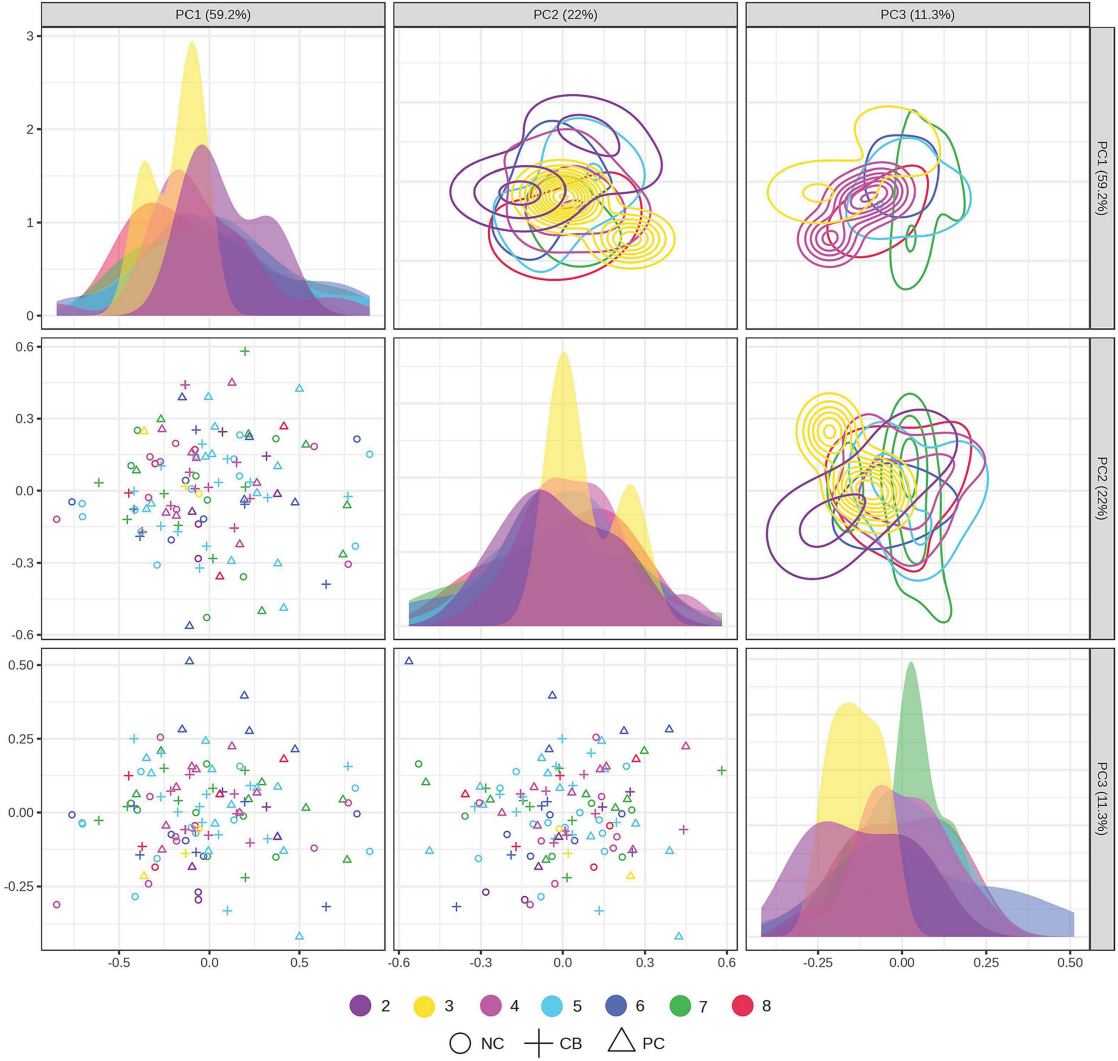
Principal component analysis (PCA) scores plot projected in the two-dimensional matrices of samples grouped by treatment and polygenic score. Participants (*n* = 37) were randomized and double-blinded to receive a standardized meal containing 600 mg choline either as choline bitartrate (CB) or phosphatidylcholine (PC), or no choline (NC) control, with a 1-week washout break in between. Single nucleotide polymorphisms (SNPs) were coded as 0, 1, or 2, representing the number of variant alleles (0 having no copy of the SNP; 1 having one copy of the SNP; and 2 having two copies of the SNP) for *choline dehydrogenase* (*CHDH*) rs12676, *betaine:homocysteine methyltransferase* (*BHMT*) rs3733890, *choline kinase A* (*CHKA*) rs10791957, and *phosphatidylethanolamine N-methyltransferase* (*PEMT*) rs4646343. Then the polygenic score was derived by summing the number of copies of the SNP. The data were log-transformed and range-scaled (mean-centered and divided by the range of each variable). The scores plot shows that 59.2% of the variation was explained by the first principal component, 22% of the variation was explained by the second component, and 11.3% of the variation was explained by the third component. No distinctive patterns were observed across treatments and polygenic scores (not significant by PERMANOVA). Different symbols denote different treatment groups for CB, PC, or NC control, whereas different colors denote different groups of polygenic scores from 2 to 8.

**Figure 6:**
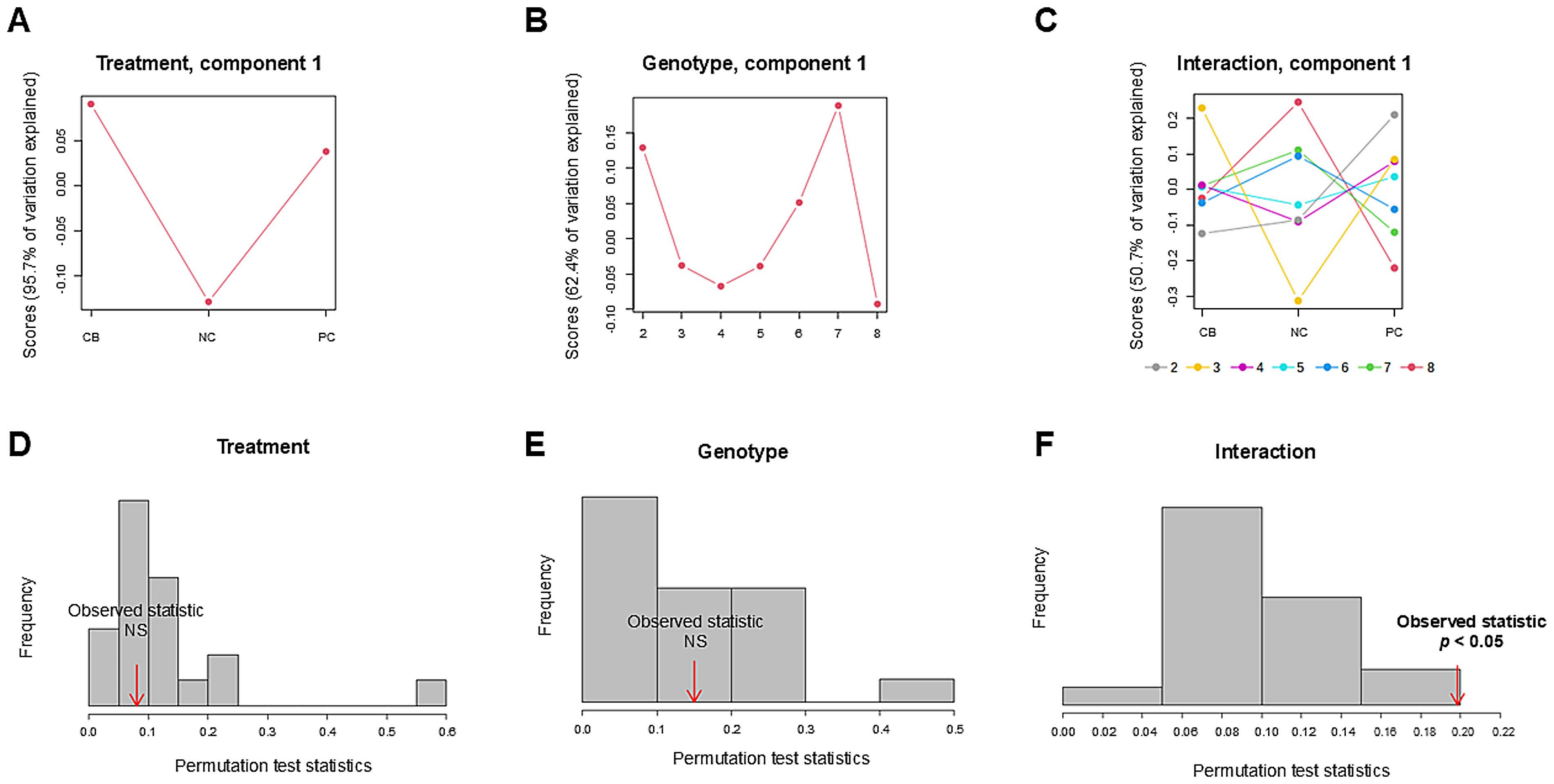
Analysis of variance simultaneous component analysis (ASCA) of treatment, genotype, and their interaction. Participants (*n* = 37) were randomized and double-blinded to receive a standardized meal containing 600 mg choline either as choline bitartrate (CB) or phosphatidylcholine (PC), or no choline (NC) control, with a 1-week washout break in between. To capture the cumulative effect of genotypes, single-nucleotide polymorphisms (SNPs) were coded as 0, 1 or 2, representing the number of variant alleles (0 having no copy of the SNP; 1 having one copy of the SNP; and 2 having two copies of the SNP) for *choline dehydrogenase* (*CHDH*) rs12676, *betaine:homocysteine methyltransferase* (*BHMT*) rs3733890, *choline kinase A* (*CHKA*) rs10791957 and *phosphatidylethanolamine N-methyltransferase* (*PEMT*) rs4646343 then the polygenic score was derived from summing the number of copies of the SNP. Sub-models of **(A)** treatment, **(B)** genotype, and **(C)** interaction were projected to identify major patterns associated with each factor, as computed scores based on the first component. The first component explained 95.7% of the variation for treatment, 62.4% of the variation for genotype, and 50.7% of the variation for the interaction between treatment and genotype in the respective ASCA sub-models. Within the interaction sub-model, the groups with the polygenic scores of 2–5 (less accumulated number of effect alleles) showed divergent patterns compared to the groups with the polygenic scores of 6–8 (more accumulated number of effect alleles) wherein the polygenic scores of 6–8 deviated from the pattern derived from treatment alone for CB, NC, and PC but not with the polygenic scores of 2–5. Model validation is shown as histograms of the distribution formed by 100 permutations. The total sum of squares for **(D)** treatment, **(E)** genotype, and **(F)** interaction was compared against that from the permuted data. Significance indicates the observed sum of squares exceeding the 95th percentile of the permuted distribution. Only the interaction term was significant, indicating the importance of both treatment and genotype factors in shaping the patterns of metabolite response to different forms of choline. The data were log-transformed and range-scaled (mean-centered and divided by the range of each variable). NS denotes not significant.

The PCA scores plot demonstrated that the first three components explained 59.2, 22, and 11.3% variation in the order of components 1, 2, and 3 without distinctive patterns across treatments or polygenic scores (Figure 5). We leveraged ASCA to model genotype and treatment effects with an interaction. The scree plots (Supplementary Figure 1) showed a clear separation among the principal components contributing to the total variation in each dataset, with the first component showing the highest variation explained. Using the first component, sub-models were created for treatment, genotype, and interaction to identify major patterns associated with each factor (Figures 6A–C). The first component explained 95.7% of the variation for treatment, 62.4% for the genotype, and 50.7% for the interaction in the respective ASCA sub-models. Treatment and genotype (grouped by polygenic score) led to distinctive patterns in sub-models through ASCA. Within the interaction sub-model, there was a divergence in patterns in the groups with the polygenic scores of 2–5 (less accumulated number of effect alleles) versus groups with the polygenic scores of 6–8 (more accumulated number of effect alleles). The pattern from the polygenic scores of 2–5 aligned with the pattern derived from treatment alone for CB, NC, and PC. Contrasting this, there was a misalignment with the polygenic scores of 6–8 away from the pattern derived from treatment alone for CB, NC, and PC. Sub-models of treatment, genotype, and interaction were validated through 100 permutations, where the total sum of squares for treatment, genotype, and interaction was compared against that from the permuted data and shown as the histogram of the distribution (Figures 6D–F). The separation distance between the original and permuted samples, as determined by permutation statistics, did not show significance for treatment alone nor genotype alone. However, the interaction term was significant with *p* < 0.05, indicating that both treatment and genotype were important predictors of metabolite responses to CB and PC.

## 4 Discussion

The findings from this study support our hypothesis that targeted SNPs in genes involved in choline homeostasis are determinants of one-carbon metabolite responses following consumption of supplemental choline differing in form. Several key findings were uncovered: (1) a main treatment effect was detected for urinary choline change from baseline reflective of differences in metabolism afforded by water-soluble versus lipid-soluble choline; (2) genetic variation in *CHDH* rs12676 was manifested as lower choline oxidation and downstream pathways in the methionine cycle in response to CB; (3) metabolic inefficiencies were observed with genetic variation in *BHMT* rs3733890 that led to lower utilization of dimethylglycine in response to PC; (4) genetic variations in *CHKA* rs10791957 and *PEMT* rs4646343 were manifested as disturbances in choline phosphorylation and synthesis with differences in the partitioning of choline in response to CB and PC; and (5) multivariate analysis showed that groups with accumulated number of effect alleles possessed contrasting responses to CB and PC, deviating from the patterns derived from the treatment effect alone. Overall, we build on our ongoing endeavor investigating metabolic pathways involving choline as a versatile compound with a wide range of biological functions (6), and present novel evidence that the complexity of individual responses arises with different forms of choline that can be distinguished by common genetic polymorphisms.

The main treatment effect, wherein a higher change in urinary choline was found with CB supplementation compared to NC control, without differences between PC compared to either NC control or CB, reflects the contribution of solubility to choline metabolism. Choline salts, including CB, are common constituents of dietary supplements, whereas PC is found in various animal and plant source foods. Free choline is characterized by more rapid absorption as opposed to PC, which must first be cleaved by pancreatic phospholipase A2 IB to release its choline moiety prior to absorption (38). These differences may explain a slower turnover of choline from PC as opposed to CB in our study and are in agreement with other reports of delayed increase in plasma choline concentrations upon PC supplementation and not with water-soluble choline (39, 40). Choline assimilation from the lipidic choline pool may be beneficial in achieving a balanced increase (40), where the reliance on PC to recycle choline may occur depending on the availability of free choline (25). Together, variations in choline responses may arise from multiple mechanisms and/or pathways to support cellular demand, highlighting the complexity of metabolic regulation, thus warranting a closer examination of choline utilization and redistribution.

No other main effects of the treatment were observed outside of choline, but we detected an interaction effect between treatment and *CHDH* rs12676 genotype that arose with CB but not with PC. CHDH catalyzes the initial step in the oxidation of choline to betaine, and genetic variation in *CHDH* has been implicated in metabolic disorders (41). We found that the AA+AC genotype group of *CHDH* rs12676 had higher urinary change from baseline for choline, betaine, dimethylglycine, and methionine upon CB supplementation compared to the NC control, but these elevations did not occur in the CC genotype group. Supporting the interaction effect, lower urinary change from baseline for betaine, dimethylglycine, and methionine were found after CB consumption in the CC genotype group compared to the AA + AC genotype group, indicating lower capacity through the methionine cycle. This suggests that the *CHDH* rs12676 polymorphism may impair choline oxidation, thereby limiting betaine availability and downstream methylation reactions. Our study expands on prior evidence of higher betaine across water-soluble and lipid-soluble choline supplementation (39), which our study provides as a dimension of genotype in influencing choline and downstream metabolites, but we found no interaction effect with PC supplementation across the genotype groups of *CHDH* rs12676. Sample type and nutrient–nutrient interaction in different study designs may have contributed to distinctive metabolic outcomes, as with other genotypes that can govern the complex nature of choline homeostasis.

One marker that emerged with PC supplementation was an interaction between treatment and *BHMT* rs3733890 genotype, which did not arise with CB. BHMT catalyzes the transfer of a methyl group from betaine to homocysteine, forming dimethylglycine and methionine, which maintains the methionine cycle and supports cellular methylation capacity (42). The GG + GA genotype group of *BHMT* rs3733890 had higher urinary choline and dimethylglycine change from baseline upon PC supplementation compared to the NC control, but these effects were absent in the AA genotype group. The reduced capacity of betaine as a methyl donor was also observed in the AA genotype group compared to the GG + GA genotype group and aligns with impaired responses to PC, both upstream and downstream (choline and dimethylglycine). This suggests that the *BHMT* rs3733890 polymorphism may reduce enzyme efficiency, leading to shifts in choline metabolite flux. Previously, a lack of association between the *BHMT* rs3733890 genotype and susceptibility to choline deficiency (9) has been reported, which was attributed to the protein product of the gene variant that did not differ in either catalytic activity or betaine binding compared to the enzyme without the polymorphism (43, 44). However, the variant is known to have a functional role in choline dynamics (13) as a metabolic node between betaine and CDP-derived PC endpoints. CB supplementation can be juxtaposed as seen with higher urinary choline change from baseline compared to the NC control in the AA genotype group of *BHMT* rs3733890. Lower flux through BHMT can be reflected as an accumulation of choline, but the GG + GA genotype group also had higher choline with CB supplementation. Higher concentration of choline without elevation in the product of the BHMT reaction in these individuals may indicate that CB favors the entry into the CDP–choline pathway rather than the methionine cycle. Methionine did not differ across genotype and treatment, but similar to another report, the loss of BHMT-catalyzed biosynthesis may not emerge with the presence of a larger methionine pool (45), although downstream transmethylation reactions would need to be investigated.

Subtle effects of urinary choline change from baseline occurred with the *CHKA* rs10791957 and *PEMT* rs4646343 genotypes when CB or PC was provided. PC synthesis occurs via two main pathways: the CDP-choline pathway, which begins with the phosphorylation of choline by CHKA (14), and the *de novo* pathway, where phosphatidylethanolamine undergoes triple methylation reactions catalyzed by PEMT (15, 16). *CHKA* rs10791957 and *PEMT* rs4646343 are located within the first intron region of the respective gene that may play a regulatory role as an enhancer of transcription levels (46). The presence of the effect alleles of *CHKA* rs10791957 is thought to confer protection from low-choline-associated organ dysfunction (17), in contrast to increased risk of low-choline-associated organ dysfunction with the effect alleles of *PEMT* rs4646343 (47). In the paradigm of extra choline, higher urinary choline change from baseline was found in the AA genotype group of *CHKA* rs10791957 after CB supplementation compared to the NC control, without alterations in betaine, dimethylglycine, and methionine, which may reflect enhanced recycling of dietary choline (choline → PC → choline). We also observed higher choline change from baseline after CB and PC supplementation compared to NC control in the CC + CA genotype group of *CHKA* rs10791957 and the GG + GT genotype group of *PEMT* rs4646343, an effect that appears to be driven by choline treatment through the intact pathways of PC synthesis. Urinary choline change from baseline did not differ in the TT genotype group of *PEMT* rs4646343 upon CB or PC supplementation compared to NC control, which may indicate lower flux of choline despite choline provision. Past studies, albeit using other designs and populations, have demonstrated the partitioning of choline toward the maintenance of PC under various physiological states (48, 49). The current study yields further nuance to the effect of the alleles of genes involved in PC synthesis in explaining metabolic heterogeneity in response to CB or PC.

Our findings complement those from Ganz et al. (10), reporting the influence of genetic variation on choline partitioning and methyl donor utilization in healthy third-trimester pregnant, lactating, and non-pregnant women consuming choline at or above current recommendations. Given the distinct research questions, the study designs of Ganz et al. and ours diverged substantially; thus, any direct comparisons should be interpreted within the context of each study. For *CHDH* rs12676, Ganz et al. showed that variants appear to favor the use of dietary choline for PEMT-PC synthesis relative to CDP-PC, whereas our study demonstrated that the CC genotype group exhibited lower urinary change for betaine, dimethylglycine, and methionine in response to CB, with no effects following PC. For *BHMT* rs3733890, Ganz et al. reported a potential preferential use of dietary choline for CDP-PC synthesis, partitioning away from betaine synthesis, where our study supports a genotype-dependent response to PC, with the AA group showing reduced methyl donor utilization. *CHKA* rs10791957 variants were implicated in decreased use of dietary choline for PEMT-PC synthesis relative to CDP-PC synthesis in Ganz et al., and our study linked the AA genotype to enhanced recycling of dietary choline, as evidenced by elevated urinary choline following CB supplementation. Ganz et al. noted that *PEMT* rs4646343 variants had lower PEMT-PC/CDP-PC, indicative of lower PEMT activity, and our data showed that individuals with the GG + GT genotype had higher urinary choline following CB and PC supplementation, whereas the TT genotype group showed no response, suggesting impaired PC synthesis. Together, both studies identified key SNPs as potential modulators of choline metabolism in various settings, with our findings offering choline form-specific insights into gene–nutrient interactions.

This study leveraged a multivariate approach incorporating the interaction between treatment and genotype, which illuminated that genotype clusters having polygenic scores of 6–8 deviated from the pattern derived from choline treatment. We grouped individuals based on the summation of the presence of variant alleles in line with the conceptual importance of examining cumulative effects, which a single SNP (and even a single gene) is unlikely to account for the complex phenotypes (50). In support of this, distinctive structures were absent in our classical PCA when only the individual factors were considered, but it became apparent once an interaction term was included through ASCA (36). We elected to employ ASCA based on the ability to calculate the permuted variation associated with each factor and interaction. This approach has the advantage of decomposing principal components separately and performing PCA on each partition that can further be validated. Based on the first component, which explained the largest percent variation, the divergence between the polygenic scores of 6–8 (with the accumulated risk alleles) versus 2–5 suggests the importance of all four SNPs (*CHDH* rs12676, *BHMT* rs3733890, *CHKA* rs10791957, and *PEMT* rs4646343) in dictating subgroups of individuals who are less responsive to CB and PC. The overall patterns, revealed through ASCA, offer utility in detecting unique responses to choline treatment that may extend to functional relevance for individual phenotype. Sub-model validations through permutations also reinforced that the interaction term (not individual factors alone) was a significant determinant of differential metabolic responses. Although not comparable, our results are concurrent with other studies that show value in grouping SNPs from similar metabolic pathways to allow for the identification of shared SNP signatures in explaining hepatic steatosis (51, 52). These types of integration can serve as an initial step toward the larger goal of identifying sources of heterogeneity to understand meaningful patterns between SNPs and health (2). We also acknowledge that there may be effects on the activity of the same enzyme from multiple SNPs, which a variant at one locus may manifest its effect when a variant at another locus is present (53). Thus, the development of algorithms to capture these interactions will clarify how multiple hits from genetic variations contribute to perturbations in metabolic pathways.

The implication of our research is that the future of precision nutrition holds promise in deciphering complex patterns of genetic variations to predict metabolic inefficiencies. Select SNPs can be grounded in a hypothesis-driven approach within nutrient-specific metabolic pathways, and this study focused on those that influence choline metabolism. As various forms of choline exist that can be obtained exogenously, SNPs alone without considering components of choline, or treatment alone without considering SNPs, would not suffice in identifying meaningful patterns of metabolic responses. Our study suggests that the stimulation of choline oxidation, use of the methionine cycle, and choline synthesis can be triggered with CB or PC in a genotype-dependent manner. The accumulated presence of the variant alleles across the select SNPs may render individuals to be less responsive to CB or PC, which may necessitate strategies to restore choline homeostasis. When different groups of individuals can be stratified on the basis of biomarkers of metabolism, better estimates of their nutrient requirements can be made, which would yield broader translational relevance to refine dietary interventions and guidelines. These outlooks, combined with the use of computational tools, can then parse individual characteristics. Extending further, the ultimate goal of precision nutrition would comprise mechanistic and functional evidence of the gene that can positively influence the guidance of clinical practice and dietary recommendations (54), including those centered on choline, thus may help optimize health in various subgroups of the population.

There are many limitations in this study. First, the genotype distribution was not equal since it was a *post hoc* design, and the modest sample size (*n* = 37) may have limited the ability to detect subtle genotype–treatment interactions, particularly when stratified by genotype and treatment groups. Although our study had utility in informing future studies to target SNPs, larger analyses are needed to validate our findings. Second, our final participant pool included only adult males and was mostly comprised of those of European descent, limiting the generalizability of our findings to other subgroups of the population. Of note, premenopausal adult women have a lower requirement for choline because of estrogen-mediated induction of the *PEMT* gene that enables endogenous biosynthesis of the choline moiety (47). The effect of SNPs, including *PEMT* rs4646343, can be regulated by estrogen, which may impact metabolic responses to CB or PC. The disparate frequency of functional variants in choline metabolism has also been found in racial and ethnic groups (17), with the existence of dietary selective pressures (55); thus, broader genetic patterns in relation to metabolic outcomes need to be further studied. Finally, this study specifically focused on choline and downstream metabolites, but other pathways can influence choline availability, including the gut microbial conversion of choline to trimethylamine and subsequent oxidation to trimethylamine-*N*-oxide (TMAO) (56). We have previously shown differential TMAO response to a meal challenge containing choline and other substrates and found that the elevation in TMAO was a function of the gut microbiota composition and genotype (27, 28, 57). Studies that link one-carbon metabolism with an integrative analysis that includes host and gut microbiota factors would provide additional insights into metabolic heterogeneity.

In conclusion, genetic variations in key genes regulating choline metabolism (*CHDH*, *BHMT*, *CHKA,* and *PEMT*) modulate metabolite profiles across distinct biochemical pathways, reflecting individualized metabolic responses to different forms of choline supplementation. Genetic variation in *CHDH* rs12676 impaired choline oxidation and disrupted the methionine cycle specifically in response to CB, while the metabolic effects of *BHMT* rs3733890 were evident only with PC supplementation. *CHKA* rs10791957 and *PEMT* rs4646343 further distinguished individuals with altered PC synthesis capacity. The combined effects of these variant alleles may diminish responsiveness to choline supplementation, grouping individuals according to their cumulative metabolic perturbations. Collectively, considering genetic variations and nutrients together, including solubility, offers a refined understanding of metabolic heterogeneity. Our findings suggest new possibilities for advancing precision nutrition that integrates the complex interplay of gene–nutrient interactions and moving beyond the scope of conventional dietary guidelines.

## Data Availability

The original contributions presented in the study are included in the article/Supplementary material. Further inquiries can be directed to the corresponding author.

## Supplementary material

SUPPLEMENTARY FIGURE 1 Scree plots of the proportion of explained variability across principal components for each sub-model in ANOVA simultaneous component analysis: **(A)** main effect of treatment; **(B)** main effect of genotype; **(C)** interaction between treatment and genotype; **(D)** residual variation. For each sub-model, the first principal component, representing the highest proportion of explained variation, was extracted for downstream analysis.
