# Supplementary figures and images for: Common genetic polymorphisms define one-carbon metabolite responses to different forms of choline in healthy adult males
Source: Front Nutr. 2025 Dec 3;12:1620538. doi: 10.3389/fnut.2025.1620538 (PMC12708588; doi:10.3389/fnut.2025.1620538)

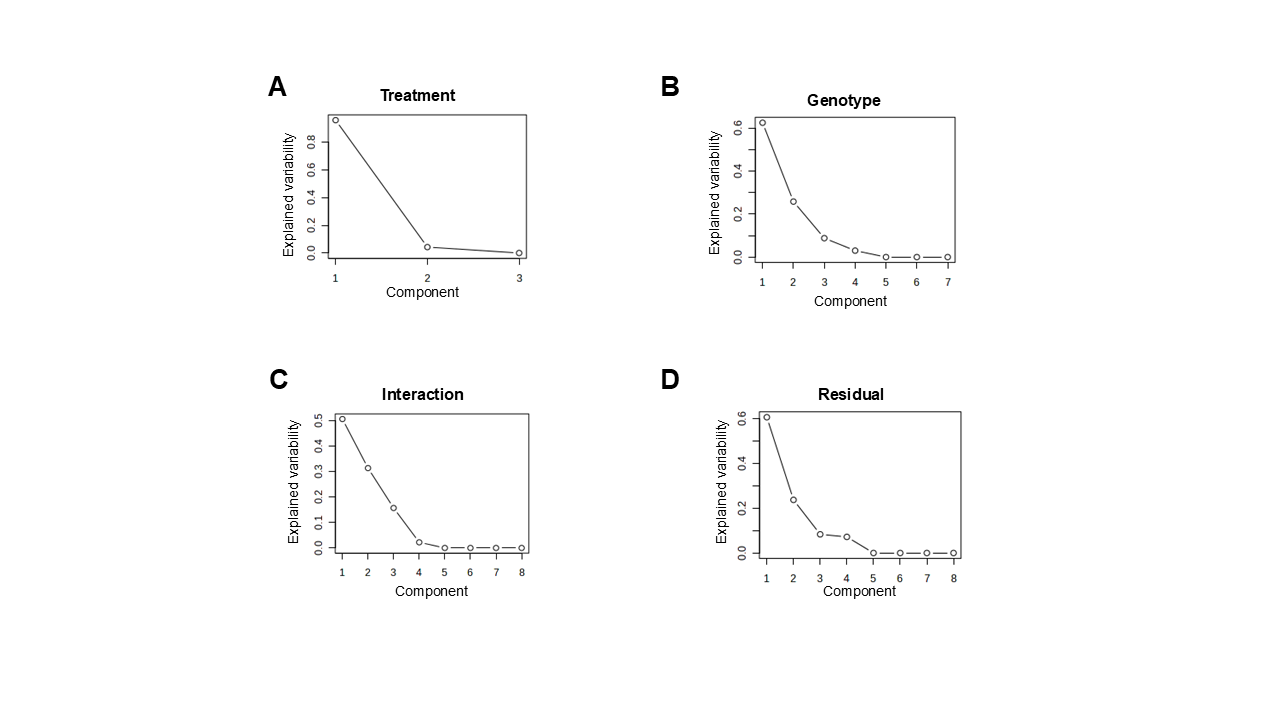

Supplement: SUPPLEMENTARY FIGURE 1 — Scree plots of the proportion of explained variability across principal components for each sub-model in ANOVA simultaneous component analysis: (A) main effect of treatment; (B) main effect of genotype; (C) interaction between treatment and genotype; (D) residual variation. For each sub-model, the first principal component, representing the highest proportion of explained variation, was extracted for downstream analysis. [file Image_1.TIF]
