# Supplementary material for: Common genetic polymorphisms define one-carbon metabolite responses to different forms of choline in healthy adult males
Source: Front Nutr. 2025 Dec 3;12:1620538. doi: 10.3389/fnut.2025.1620538 (PMC12708588; doi:10.3389/fnut.2025.1620538)
Supplement: Supplementary file 2 [file Table_1.DOCX]

Supplementary Material

# Supplementary Table 1. Single nucleotide polymorphisms of focus in this study. Abbreviations: *choline dehydrogenase* (*CHDH*), *betaine:homocysteine methyltransferase* (*BHMT*), *choline kinase A* (*CHKA*), *phosphatidylethanolamine N-methyltransferase* (*PEMT*).

| **Gene** | **ID** | **Alleles** | **Assay** |
| --- | --- | --- | --- |
| *CHDH* | rs12676 | A>C | C__7553898_10 |
| *BHMT* | rs3733890 | G>A | C__11646606_20 |
| *CHKA* | rs10791957 | C>A | C__26899865_20 |
| *PEMT* | rs4646343 | G>T | C__9246129_10 |

# Supplementary Table 2. Urinary concentrations of choline, betaine, dimethylglycine and methionine following consumption of choline bitartrate (CB), phosphatidylcholine (PC) or no choline (NC) relative to baseline in a randomized, double-blinded crossover study. Data are stratified by *choline dehydrogenase* (*CHDH*) rs12676 genotype (AA+AC vs CC). Values represent mean, standard error of the mean (SEM) and interquartile range (25th percentile, median and 75th percentile). Sample sizes per group: AA+AC (n = 13) and CC (n = 24).

|  |  | **NC** | | **CB** | | **PC** | |
| --- | --- | --- | --- | --- | --- | --- | --- |
| ***CHDH* rs12676** |  | **AA+AC**  **(n = 13)** | **CC**  **(n = 24)** | **AA+AC**  **(n = 13)** | **CC**  **(n = 24)** | **AA+AC**  **(n = 13)** | **CC**  **(n = 24)** |
| Choline | Mean | 0.44 | 0.61 | 1.81 | 1.06 | 0.76 | 1.04 |
|  | SEM | 0.15 | 0.11 | 0.33 | 0.18 | 0.15 | 0.13 |
|  | 25% | 0.05 | 0.23 | 0.92 | 0.25 | 0.52 | 0.47 |
|  | Median | 0.19 | 0.48 | 1.25 | 1.05 | 0.86 | 0.96 |
|  | 75% | 0.95 | 0.84 | 2.36 | 1.61 | 1.08 | 1.44 |
| Betaine | Mean | 4.32 | 3.99 | 7.97 | 3.77 | 4.85 | 4.75 |
|  | SEM | 0.80 | 0.44 | 0.95 | 0.54 | 0.82 | 0.51 |
|  | 25% | 1.30 | 2.36 | 5.55 | 1.83 | 2.39 | 2.57 |
|  | Median | 4.84 | 3.89 | 6.57 | 4.03 | 3.55 | 4.65 |
|  | 75% | 5.79 | 5.20 | 11.38 | 4.82 | 7.36 | 6.91 |
| Dimethylglycine | Mean | 1.72 | 1.72 | 3.08 | 1.69 | 2.49 | 2.23 |
|  | SEM | 0.37 | 0.24 | 0.34 | 0.20 | 0.34 | 0.27 |
|  | 25% | 0.62 | 0.79 | 2.15 | 0.86 | 1.86 | 1.10 |
|  | Median | 1.62 | 1.40 | 2.77 | 1.53 | 2.68 | 1.94 |
|  | 75% | 2.78 | 2.60 | 4.05 | 2.46 | 2.99 | 2.96 |
| Methionine | Mean | 1.84 | 2.06 | 3.20 | 1.77 | 2.48 | 1.84 |
|  | SEM | 0.53 | 0.33 | 0.37 | 0.22 | 0.44 | 0.19 |
|  | 25% | 0.34 | 0.65 | 2.80 | 0.91 | 1.10 | 1.17 |
|  | Median | 1.58 | 2.13 | 3.50 | 1.75 | 2.29 | 1.89 |
|  | 75% | 3.88 | 2.99 | 3.83 | 2.40 | 3.53 | 2.53 |

# Supplementary Table 3. Urinary concentrations of choline, betaine, dimethylglycine and methionine following consumption of choline bitartrate (CB), phosphatidylcholine (PC) or no choline (NC) relative to baseline in a randomized, double-blinded crossover study. Data are stratified by *betaine:homocysteine methyltransferase* (*BHMT*) rs3733890 genotype (GG+GA vs AA). Values represent mean, standard error of the mean (SEM) and interquartile range (25th percentile, median and 75th percentile). Sample sizes per group: GG+GA (n = 21) and AA (n = 16).

|  |  | **NC** | | **CB** | | **PC** | |
| --- | --- | --- | --- | --- | --- | --- | --- |
| ***BHMT* rs3733890** |  | **GG+GA**  **(n = 21)** | **AA**  **(n = 16)** | **GG+GA**  **(n = 21)** | **AA**  **(n = 16)** | **GG+GA**  **(n = 21)** | **AA**  **(n = 16)** |
| Choline | Mean | 0.42 | 0.72 | 1.16 | 1.55 | 1.07 | 0.78 |
|  | SEM | 0.08 | 0.17 | 0.18 | 0.32 | 0.14 | 0.14 |
|  | 25% | 0.13 | 0.28 | 0.65 | 0.31 | 0.57 | 0.46 |
|  | Median | 0.27 | 0.69 | 1.04 | 1.34 | 0.96 | 0.76 |
|  | 75% | 0.72 | 1.29 | 1.52 | 2.49 | 1.55 | 1.24 |
| Betaine | Mean | 3.75 | 4.58 | 4.95 | 5.63 | 5.82 | 3.43 |
|  | SEM | 0.59 | 0.48 | 0.64 | 1.07 | 0.64 | 0.32 |
|  | 25% | 1.81 | 2.83 | 3.43 | 3.08 | 3.21 | 2.46 |
|  | Median | 2.52 | 4.30 | 4.32 | 4.60 | 7.05 | 3.21 |
|  | 75% | 5.45 | 5.48 | 6.99 | 7.67 | 8.19 | 4.66 |
| Dimethylglycine | Mean | 1.64 | 1.82 | 2.13 | 2.24 | 2.74 | 1.76 |
|  | SEM | 0.31 | 0.23 | 0.28 | 0.32 | 0.26 | 0.30 |
|  | 25% | 0.60 | 1.20 | 1.37 | 1.39 | 1.94 | 0.87 |
|  | Median | 1.30 | 1.69 | 1.91 | 2.54 | 2.50 | 1.50 |
|  | 75% | 2.89 | 2.46 | 3.07 | 2.79 | 3.47 | 2.90 |
| Methionine | Mean | 2.11 | 1.82 | 2.41 | 2.09 | 2.28 | 1.79 |
|  | SEM | 0.40 | 0.38 | 0.29 | 0.36 | 0.26 | 0.33 |
|  | 25% | 0.44 | 0.61 | 1.41 | 0.94 | 1.58 | 0.86 |
|  | Median | 2.21 | 1.70 | 2.43 | 1.89 | 2.22 | 1.53 |
|  | 75% | 3.88 | 2.61 | 3.42 | 3.42 | 3.06 | 2.42 |

# Supplementary Table 4. Urinary concentrations of choline, betaine, dimethylglycine and methionine following consumption of choline bitartrate (CB), phosphatidylcholine (PC) or no choline (NC) relative to baseline in a randomized, double-blinded crossover study. Data are stratified by *choline kinase A* (*CHKA*) rs10791957 genotype (CC+CA vs AA). Values represent mean, standard error of the mean (SEM) and interquartile range (25th percentile, median and 75th percentile). Sample sizes per group: CC+CA (n = 25) and AA (n = 12).

|  |  | **NC** | | **CB** | | **PC** | |
| --- | --- | --- | --- | --- | --- | --- | --- |
| ***CHKA* rs10791957** |  | **CC+CA**  **(n = 25)** | **AA**  **(n = 12)** | **CC+CA**  **(n = 25)** | **AA**  **(n = 12)** | **CC+CA**  **(n = 25)** | **AA**  **(n = 12)** |
| Choline | Mean | 0.41 | 0.83 | 1.09 | 1.83 | 1.01 | 0.82 |
|  | SEM | 0.08 | 0.20 | 0.16 | 0.39 | 0.11 | 0.21 |
|  | 25% | 0.19 | 0.18 | 0.38 | 0.85 | 0.55 | 0.29 |
|  | Median | 0.28 | 0.88 | 1.06 | 1.33 | 0.91 | 0.76 |
|  | 75% | 0.72 | 1.42 | 1.70 | 2.68 | 1.38 | 1.15 |
| Betaine | Mean | 4.31 | 3.69 | 5.05 | 5.64 | 4.92 | 4.51 |
|  | SEM | 0.53 | 0.52 | 0.65 | 1.21 | 0.53 | 0.77 |
|  | 25% | 2.25 | 1.93 | 2.73 | 3.92 | 2.86 | 2.19 |
|  | Median | 4.19 | 4.11 | 4.86 | 4.42 | 4.62 | 3.95 |
|  | 75% | 6.19 | 5.38 | 7.56 | 6.19 | 7.35 | 6.50 |
| Dimethylglycine | Mean | 1.82 | 1.51 | 2.03 | 2.48 | 2.45 | 2.04 |
|  | SEM | 0.25 | 0.34 | 0.28 | 0.26 | 0.26 | 0.34 |
|  | 25% | 0.75 | 0.65 | 1.07 | 1.69 | 1.53 | 1.11 |
|  | Median | 1.56 | 1.32 | 1.91 | 2.54 | 2.36 | 2.19 |
|  | 75% | 2.78 | 2.39 | 2.74 | 3.09 | 3.34 | 2.77 |
| Methionine | Mean | 1.80 | 2.36 | 2.09 | 2.64 | 1.92 | 2.38 |
|  | SEM | 0.33 | 0.52 | 0.28 | 0.36 | 0.24 | 0.38 |
|  | 25% | 0.37 | 0.65 | 1.26 | 1.67 | 1.10 | 1.68 |
|  | Median | 2.05 | 1.80 | 2.09 | 3.01 | 1.90 | 2.34 |
|  | 75% | 2.88 | 4.24 | 3.06 | 3.65 | 2.42 | 3.03 |

# Supplementary Table 5. Urinary concentrations of choline, betaine, dimethylglycine and methionine following consumption of choline bitartrate (CB), phosphatidylcholine (PC) or no choline (NC) relative to baseline in a randomized, double-blinded crossover study. Data are stratified by *phosphatidylethanolamine N-methyltransferase* (*PEMT*) rs4646343 genotype (GG+GT vs TT). Values represent mean, standard error of the mean (SEM) and interquartile range (25th percentile, median and 75th percentile). Sample sizes per group: GG+GT (n = 28) and TT (n = 9).

|  |  | **NC** | | **CB** | | **PC** | |
| --- | --- | --- | --- | --- | --- | --- | --- |
| ***PEMT* rs4646343** |  | **GG+GT**  **(n = 28)** | **TT**  **(n = 9)** | **GG+GT**  **(n = 28)** | **TT**  **(n = 9)** | **GG+GT**  **(n = 28)** | **TT**  **(n = 9)** |
| Choline | Mean | 0.51 | 0.68 | 1.33 | 1.31 | 1.08 | 0.51 |
|  | SEM | 0.10 | 0.17 | 0.19 | 0.40 | 0.12 | 0.09 |
|  | 25% | 0.09 | 0.24 | 0.78 | 0.24 | 0.63 | 0.25 |
|  | Median | 0.35 | 0.57 | 1.06 | 1.06 | 1.05 | 0.45 |
|  | 75% | 0.90 | 0.89 | 1.83 | 2.62 | 1.49 | 0.78 |
| Betaine | Mean | 3.97 | 4.55 | 5.62 | 4.06 | 4.89 | 4.45 |
|  | SEM | 0.41 | 1.06 | 0.71 | 0.89 | 0.50 | 0.90 |
|  | 25% | 2.36 | 1.92 | 3.93 | 1.99 | 2.63 | 2.32 |
|  | Median | 4.08 | 4.33 | 4.77 | 3.90 | 4.72 | 3.21 |
|  | 75% | 5.38 | 7.65 | 7.64 | 5.88 | 7.11 | 7.60 |
| Dimethylglycine | Mean | 1.62 | 2.02 | 2.33 | 1.70 | 2.33 | 2.30 |
|  | SEM | 0.21 | 0.49 | 0.24 | 0.38 | 0.25 | 0.38 |
|  | 25% | 0.65 | 1.04 | 1.47 | 0.94 | 1.32 | 1.41 |
|  | Median | 1.38 | 2.03 | 2.41 | 1.43 | 2.20 | 2.46 |
|  | 75% | 2.60 | 3.24 | 2.98 | 2.75 | 3.02 | 2.72 |
| Methionine | Mean | 2.04 | 1.80 | 2.44 | 1.75 | 1.92 | 2.52 |
|  | SEM | 0.33 | 0.52 | 0.27 | 0.32 | 0.25 | 0.28 |
|  | 25% | 0.41 | 1.10 | 1.35 | 1.11 | 1.10 | 1.95 |
|  | Median | 2.02 | 1.77 | 2.37 | 1.67 | 1.82 | 2.54 |
|  | 75% | 3.52 | 2.93 | 3.71 | 2.65 | 2.45 | 3.07 |

# Supplementary Figure 1. Scree plots of the proportion of explained variability across principal components for each sub-model in ANOVA simultaneous component analysis: (A) main effect of treatment; (B) main effect of genotype; (C) interaction between treatment and genotype; (D) residual variation. For each sub-model, the first principal component, representing the highest proportion of explained variation, was extracted for downstream analysis.
